# Assessing probabilistic modelling for wind speed from numerical weather prediction model and observation in the Arctic

**DOI:** 10.1038/s41598-021-87299-4

**Published:** 2021-04-07

**Authors:** Hao Chen, Yngve Birkelund, Stian Normann Anfinsen, Reidar Staupe-Delgado, Fuqing Yuan

**Affiliations:** 1 Department of Technology and Safety, UiT The Arctic University of Norway, 9019 Tromsø, Norway; 2 Department of Physics and Technology, UiT The Arctic University of Norway, 9019 Tromsø, Norway

**Keywords:** Energy science and technology, Renewable energy, Wind energy

## Abstract

Mapping Arctic renewable energy resources, particularly wind, is important to ensure the transition into renewable energy in this environmentally vulnerable region. The statistical characterisation of wind is critical for effectively assessing energy potential and planning wind park sites and is, therefore, an important input for wind power policymaking. In this article, different probability density functions are used to model wind speed for five wind parks in the Norwegian Arctic region. A comparison between wind speed data from numerical weather prediction models and measurements is made, and a probability analysis for the wind speed interval corresponding to the rated power, which is largely absent in the existing literature, is presented. The results of the present study suggest that no single probability function outperforms across all scenarios. However, some differences emerged from the models when applied to different wind parks. The Nakagami and Generalised extreme value distributions were chosen for the numerical weather predicted prediction and the observed wind speed modelling, respectively, due to their superiority and stability compared with other methods. This paper, therefore, provides a novel direction for understanding the numerical weather prediction wind model and shows that its speed statistical features are better captured than those of real wind.

## Introduction

With the growing reliance on renewable energy resources in many regions of the world, studying the predictability of renewable energy is becoming progressively important^1^. As one of the cleanest renewable energy sources, wind energy has attracted growing attention worldwide^2^. In Norway, multiple wind energy projects have been developed for energy markets, and many more wind parks are in the design and planning stage. It is, therefore, essential to create a compelling and effective method for evaluating wind energy resources in the region. Accurately assessing local wind energy potential and resources is a crucial part of wind energy development and enhances investor confidence in financial feasibility and risk acceptability^3^. Wind resource potential varies considerably from one wind park site to another due to geographical and topographical differences. Therefore, an accurate assessment of a wind park's wind energy potential is necessary when developing sustainable wind power projects^4^. A rigorous evaluation of the potential wind speed resources of a specific location directly affects the economic value, risk assessment, turbine selection, power generation estimation of the wind park, as well as the operation and management of wind power conversion systems^5^. In other words, proper attention to site selection is crucial for long-term sustainability gains in wind power investments, in addition to social priorities due to the recognised nuisance conflicts that have previously arisen in the context of wind power developments.

Since wind speed is variable, intermittent and uncertain, appropriate means should be used to describe its fluctuating nature^6^. The probability density function (PDF) and the related cumulative distribution function (CDF) are often used in wind resource assessments to quantify the theoretical wind energy potential of an area. Both of them intuitively reflect the statistical characteristics of wind speed. Wind is created by pressure differences between different regions, but terrain features like mountains, valleys, fjords and other surface irregularities create disturbances, meaning that wind speeds near the ground typically fluctuate significantly. The wind speed contributing to energy production in a wind turbine surrounded by complex terrain typically changes significantly; therefore, when the time scale is short, the statistical characteristics of the wind become uncertain and difficult to predict^7^. When the time scale is long, the probabilistic distribution of wind speed is relatively stable, and the long-term statistical characteristics of wind can be determined^8^. A common way of describing the wind energy at a site is to use its annual wind speed distribution. The PDF of wind speed is vital in valuing energy production for wind power and is an important evaluation index for estimating local wind resource potential.

### Related work

Some prior research on wind resources is based on probability distribution methods for specific regions with varying wind conditions and wind power potential. The two-parameter Weibull distribution is a widely used statistical distribution in wind engineering^9,10^; however, the fitting results are not optimal for some regions, which results in a substantial difference between the estimated annual power generation and the actual yearly power generation^5^. This suggests that distribution may not be a good representation of some wind conditions or some sites. Elsewhere, researchers have expressed concerns over the role of case studies for practical wind engineering purposes. Aries et al. conducted a case study of four sites’ wind speed with eight distribution models for four sites in Algeria and found that the Generalised extreme value and Gamma Distributions were the most reliable base on the root mean square error evaluation^11^. Wang et al. compared parametric and nonparametric models for wind speed probability distribution by taking four sites in central China as examples and showed the edge of nonparametric models^5^. Alavi et al. demonstrated that the most suitable probability distributions for evaluating wind speed were not the same based on five different measurement stations distributed in the east and south-east of Iran^12^. Ayodele et al. used the Weibull distribution to estimate the wind resource in a coastal area of South Africa with complex terrain^13^. Gualtieri et al. focused on coastal locations in Southern Italy and used the Weibull distribution extrapolating model to assess wind resource to the turbine hub height^14^. Allouhi et al. also used the Weibull distribution to describe the frequencies of actual wind data in six coastal locations in Morocco based on hourly wind speeds and directions data of 5 years between 2011 and 2015^15^. Jiménez et al. found that atmospheric stability plays a major role in controlling the shape of the wind speed distribution. The authors showed that the shape wind speed measured from a combination of long-term wind observations and numerical simulations is strongly modulated by the numerical atmospheric scales^16^.

Most studies in this field have focused on PDF modelling for the observed wind speed of wind parks, and there is a lack of PDF modelling for wind speed forecasted by Numerical Weather Prediction (NWP). This is unfortunate because NWP calculations generate the vast majority of the world's wind data. Some studies have focused on using the Weibull distribution or one of three or four other similar distribution methods. However, they fail to consider the broader deployment of the PDF approach for wind speed modelling. In practice, more attention is paid to the wind speed range corresponding to the wind turbine's rated power. Despite this, few studies have applied PDF methods to analyse wind speed intervals when wind turbines are producing the maximum power, and little research has discussed wind speed distribution in the Arctic region.

### Objectives

In this research, we comparatively assess seven different probability distributions for wind speed modelling, some of which are classical, while others have rarely been used to estimate the wind speed distribution for five wind parks in the Norwegian Arctic coastal region. To improve the understanding of differences in wind speed data from different resources, we compared wind speed distributions for a wind park with NWP and observed wind data.

The main contributions of this paper can be summarised as follows.

1. The present study is the first to conduct a PDF modelling analysis of wind speed intervals associated with wind turbine trunnion rated power, with a particular focus on differences between interval and overall wind speed modelling.
2. This paper compares wind speed distributions based on wind data from NWP and measurements. Wind speed distributions provide an intriguing and well-established approach to analyse wind speed resources, and this paper investigates their use for NWP models in the context of complex coastal terrain.
3. The present study can assist in a more detailed understanding of PDF applications in wind speed modelling, as seven ideal distributions are used to model wind speed. Moreover, it offers an insight into the potential for renewable energy utilisation in the Arctic by conducting natural resource modelling in this area, with clear implications for practice, policy and future project implementation.

The paper is organised as follows: in “Description of wind park and wind speed data”, we describe the wind data and their sites to provide the context of the study. In “Methodology”, we elaborate on the methodological aspects of the study, while “Experiment setup and evaluation” outlines the experimental process. “Results and discussion” presents the results and main implications of the study and reflects on their relevance for research and practice. The final and concluding section summarises the most important elements of the research.

## Description of wind park and wind speed data

In the present study, we focus on five wind parks in the Norwegian Arctic regions. The second and third columns of Table 1 list their locations and the site ruggedness index (RIX)^17^. The RIX is an empirical parameter for measuring the complexity of nearby terrain and is typically used in fluid modelling or in numerical weather models to indicate identify turbulence may interfere with the model results. In our case, this was based on a fraction of the area within a 2 km radius around the location with a more than 30% degree inclination and was extracted from a Norwegian mapping of wind resources.

**Table 1:** The location of wind parks and statistics of their wind speed.

Wind park	Location °N/°E	RIX	Mean (m/s)	Standard deviation (m/s)	Min (m/s)	Max (m/s)	Coefficient of variation	Skewness	Kurtosis
Nygårdsfjellet	68.504/17.879	0–5	8.096	5.038	0.032	31.481	0.622	0.775	3.815
Raggovidda	70.098/20.081	5–10	9.490	5.101	0.107	32.430	0.538	0.666	3.361
Kjøllefjord	70.769/29.094	0–5	7.900	4.213	0.080	25.508	0.533	0.704	3.453
Havøygavlen	70.922/27.268	10–20	8.335	4.434	0.097	26.926	0.532	0.709	3.359
Fakken (NWP)	71.012/24.589	5–10	6.948	3.885	0.097	33.686	0.559	1.164	5.960
Fakken (MEASURE)	71.012/24.589	5–10	7.687	4.627	0.000	35.100	0.602	1.338	5.660

### Numerical weather prediction

Scandinavian meteorological institutes use an operational numerical weather prediction **(**NWP) forecast known as the Meteorological Ensemble Prediction System (MEPS). The NWP model is a complex mathematical model of the atmosphere that divides the Earth's surface into grids^18^. The grid's spatial resolution determines how meteorological processes are simulated with different accuracy levels, which limits the quality of the forecasts. A study conducted by the Norwegian Meteorological Institute demonstrated that the higher-resolution regional NWP model did not lead to better wind power forecasts for some Norwegian wind parks^19^. Therefore, in the present study, we considered NWP data with a horizontal resolution of 2.5 km as a relatively coarse resolution in wind predictions.

### Data description

NWP wind data from the five wind parks were extracted from Norwegian Meteorological Institute's operational MEPS models. The predictions initiated at 00, 06, 12 and 18 UTC and were made available for operational use about 2 h later. The observed wind data were offered by Troms Kraft AS—the power company that operates Fakken wind park. In the present study, we combined the forecast data into a single time series with hourly wind speed data from 0:00 on 1 January 2017 to 23:00 on 31 December 2017. The year is with wind conditions of northern Norway coastline are not significantly different from the previous 15 years. Table 1 provides a summary of the overall data. The coefficient of variation is defined as the standard deviation divided by the mean.

## Methodology

### Wind energy

In wind engineering, the capacity factor (CF) is particularly useful when conducting a fast evaluation at the early design and planning stages of a wind park. Understanding the probability distribution of wind speed is essential to calculating the CF of wind parks. The CF is calculated from the average value of wind energy produced divided by the rated wind power by a wind turbine in a certain period, which may be read from the following Eqs. (1–3):

1 $$CF=\frac{{P}_{\text{ave }}}{{P}_{r}}$$

2 $$P_{{\text{ave }}} = \mathop \smallint \limits_{0}^{\infty } f\left( v \right)P\left( v \right)dv$$

3 $$P\left( v \right) = \left\{ {\begin{array}{*{20}l} {P_{r} } \hfill & {v_{r} < v \le v_{o} } \hfill \\ {P_{r } \times g\left( v \right)} \hfill & {v_{i} < v \le v_{r} } \hfill \\ 0 \hfill & {v \le v_{i} ,\;v > v_{0} } \hfill \\ \end{array} } \right.$$

where *f*(*v*) is the PDF of wind speed, which is the main target of this research *P*(*v*) reflects the turbine power curve used to describe the power fluctuations related to wind speed. *v*_*i*_, *v*_*r*_, *v*_*o*_, and *P*_*r*_ represent the cut-in speed, the rated speed, the cut-off speed, and the rated power, respectively^5,20^. The *g*(*v*) is a multiplier increasing from 0 to 1 within the interval, that depends on the wind turbine specification. A wind turbine reaches its maximum power output when the wind speed is in the interval between the rated and cut-off speed. Adequate knowledge of the wind speed interval corresponding to the wind turbine's rated power is important for ensuring the efficient and economical operation of the turbine. Therefore, aside from the wind speed distribution modelling, we also paid special attention to wind speed in this rated power interval.

### Probability distribution

Tables 2 and 3 offer brief mathematical expressions of the seven ideal probability distributions used in the present study. These distributions are defined as follows.

- Gamma distribution is a two-parameter continuous probability distribution^21^.
- Generalised extreme value distribution (GEV) is a continuous probability distribution developed with extreme value theory^22^.
- Nakagami distribution is a generalised two parameters probability distribution model proposed by Nakagami Minoru^23^. It has received extensive attention, as it can model a broad range of fading channel conditions and describe many empirical data sets^24^.
- Normal distribution, also called Gaussian distribution, is a continuous probability distribution for ideally describing a real-valued random variable^25^.
- Rayleigh distribution essentially describes the distribution of the mode of a stochastic two-dimensional vector when the two components of the vector are independent, have the same variance and are normally distributed with zero means^26,27^.
- T distribution is commonly used to estimate the mean of a small population that is normally distributed, where the standard deviation is unknown^28^.
- Weibull distribution is the theoretical basis for reliability analysis and life inspections and is widely used for describing the probability distribution of wind speed^29^.

**Table 2:** The mathematical expressions of distributions.

Distribution	PDF	Note	CDF
Gamma	$f(x;a,b)=\frac{1}{{b}^{a}\Gamma (a)}{\int }_{0}^{x}{t}^{a-1}{e}^{-\frac{t}{b}}dt$ where $$t(x)=\left\{\begin{array}{ll}{\left(1+\xi \left(\frac{x-\mu }{\sigma }\right)\right)}^{-1/\xi }& \, \xi \ne 0\\ {e}^{-(x-\mu )/\sigma }& \, \xi =0\end{array}\right.$$	a is a shape parameter b is a scale parameter and $\Gamma \left(.\right)$ is the Gamma function	$F(x)={e}^{-t(x)}$
GEV	$f\left(x;\mu ,\sigma ,\xi \right)=\frac{1}{\sigma }t(x{)}^{\xi +1}{e}^{-t(x)}$	$\mu$ is a location parameter $\sigma >0$ is a scale parameter $\xi$ is a shape parameter	$F(x;a,b)=\frac{1}{{b}^{a}\Gamma (a)}{\int }_{0}^{x}{t}^{a-1}{e}^{-\frac{t}{b}}dt$
Nakagami	$f(x;m,\Omega )=\frac{2{m}^{m}}{\Gamma (m){\Omega }^{m}}{x}^{2m-1}\mathit{exp}\left(-\frac{m}{\Omega }{x}^{2}\right),\forall x\ge 0$	$m\ge 1/2$ is a shape parameter $\Omega \ge 0$ is a spread parameter	$F(x;m,\Omega )=\frac{\gamma \left(m,\frac{m}{\Omega }{x}^{2}\right)}{\Gamma \left(m\right)}$ where $\gamma \left(.\right)$ is the Incomplete Gamma function and $\Gamma \left(.\right)$ is the Gamma function
Normal	$f(x)=\frac{1}{\sigma \sqrt{2\pi }}{e}^{-\frac{1}{2}{\left(\frac{x-\mu }{\sigma }\right)}^{2}}$	$\mu$ is the mean $\sigma$ is the standard division erf $\left(.\right)$ is the error function	$F(x)=\Phi \left(\frac{x-\mu }{\sigma }\right)=\frac{1}{2}\left[1+\mathit{erf}\left(\frac{x-\mu }{\sigma \sqrt{2}}\right)\right]$
Rayleigh	$f(x;\sigma )=\frac{x}{{\sigma }^{2}}{e}^{-{x}^{2}/\left(2{\sigma }^{2}\right)},\hspace{1em}x\ge 0$	$\sigma >0$ is a scale parameter	$F(x;\sigma )=1-{e}^{-{x}^{2}/\left(2{\sigma }^{2}\right)}$
t	$f(x;\nu )=\frac{\Gamma \left(\frac{\nu +1}{2}\right)}{\Gamma \left(\frac{\nu }{2}\right)}\frac{1}{\sqrt{\nu \pi }}\frac{1}{{\left(1+\frac{{x}^{2}}{\nu }\right)}^{\frac{\nu +1}{2}}}$	$\nu >0$ is the number of degrees of freedom and $\Gamma \left(.\right)$ is the Gamma function	$F\left( {x;\nu } \right) = \int\limits_{{ - \infty }}^{x} {\frac{{\Gamma \left( {\frac{{\nu + 1}}{2}} \right)}}{{\Gamma \left( {\frac{\nu }{2}} \right)}}\frac{1}{{\sqrt {\nu \pi } }}\frac{1}{{\left( {1 + \frac{{t^{2} }}{\nu }} \right)^{{\frac{{\nu + 1}}{2}}} }}dt}$
Weibull	$$f\left( {x;\lambda ,k} \right) = \left\{ {\begin{array}{*{20}l} {\frac{k}{\lambda }\left( {\frac{x}{\lambda }} \right)^{k - 1} e^{{ - (x/\lambda )^{k} }} } \hfill & {x \ge 0} \hfill \\ 0 \hfill & {x < 0} \hfill \\ \end{array} } \right.$$	$k >0$ is a shape parameter and $\lambda >0$ is a scale parameter	$$F\left( {x;\lambda ,k} \right) = \left\{ {\begin{array}{*{20}l} {1 - e^{{ - (x/\lambda )^{k} }} } \hfill & {x \ge 0} \hfill \\ 0 \hfill & {x < 0} \hfill \\ \end{array} } \right.$$

**Table 3:** The mean and variance expressions of distributions.

Distribution	Mean	Variance
Gamma	*ab*	*ab*^2^
GEV	$$\left\{ {\begin{array}{*{20}l} {\mu + \sigma \left( {g_{1} - 1} \right)/\xi } & {{\text{ if }}\xi \ne 0,\xi < 1} \\ {\mu + \sigma \gamma } & {{\text{ if }}\xi = 0} \\ \infty & {{\text{ if }}\xi \ge 1} \\ \end{array} } \right.$$ where $g_{k} = \Gamma \left( {1 - k\xi } \right)$, and $\gamma$ is Euler's constant	$$\left\{ {\begin{array}{*{20}l} {\sigma^{2} {{\left( {g_{2} - g_{1}^{2} } \right)} \mathord{\left/ {\vphantom {{\left( {g_{2} - g_{1}^{2} } \right)} {\xi^{2} }}} \right. \kern-\nulldelimiterspace} {\xi^{2} }}} \hfill & {{\text{if}}\;\xi \ne 0,\;\xi < \frac{1}{2}} \hfill \\ {\sigma^{2} \frac{{\pi^{2} }}{6}} \hfill & {{\text{if}}\;\xi = 0} \hfill \\ \infty \hfill & {{\text{if}}\;\xi \ge \frac{1}{2}} \hfill \\ \end{array} } \right.$$
Nakagami	$\frac{\Gamma \left(m+\frac{1}{2}\right)}{\Gamma (m)}{\left(\frac{\Omega }{m}\right)}^{1/2}$	$\Omega \left(1-\frac{1}{m}{\left(\frac{\Gamma \left(m+\frac{1}{2}\right)}{\Gamma (m)}\right)}^{2}\right)$
Normal	$\mu$	$\sigma$
Rayleigh	$\sigma \sqrt{\frac{\pi }{2}}$	$\frac{4-\pi }{2}{\sigma }^{2}$
t	0, for $\nu >1$	$\frac{\nu }{\nu -2}$, for $\nu >2$
Weibull	$\lambda \Gamma \left( {1 + \frac{1}{k}} \right)$	${\lambda }^{2}\left[\Gamma \left(1+\frac{2}{k}\right)-{\left(\Gamma \left(1+\frac{1}{k}\right)\right)}^{2}\right]$

### Parametric estimation

Parametric estimation for the PDFs of wind speed refers to the assumption that a specific probability distribution model can describe the wind speed, where the parameters of the model are estimated based on available wind speed data. Several parametric estimation methods can be used in wind engineering, including the moment method, empirical approach, graphical method and maximum likelihood method^30^. In a study comparing six methods for estimating Weibull parameters to fit wind data, the maximum likelihood method was, on the whole, shown to provide more accurate estimations than other methods in tests with both simulated and observation data^31^. Therefore, we used the maximum likelihood method to identify the parameters for all seven probability density functions in the present study. The Maximum likelihood estimation (MLE) method can be explained as follows^32^: if {X_1_, X_2_, …, X_*n*_} is an independent and identically distributed sample from a population with PDF $f\left( {x\left| {\theta_{1} , \ldots \theta_{k} } \right.} \right).$ The likelihood function is defined by Eq. (4):

4 $$L\left( {\theta \left| X \right.} \right) = L\left( {\theta_{1} , \ldots \theta_{k} \left| {x_{1} \cdots x_{n} } \right.} \right) = \prod\limits_{i = 1}^{n} {f\left( {x_{i} \left| {\theta_{1} , \ldots \theta_{k} } \right.} \right)}$$

If $L\left( {\theta \left| X \right.} \right)$ is differentiable in *θ*, then the values of *θ*_*i*_ that minimize $L\left( {\theta_{1} , \ldots \theta_{k} \left| {x_{1} \cdots x_{n} } \right.} \right)$. are solutions of possible candidates *θ*_*i*_ for the MLE are calculated by Eq. (5):

5 $$\frac{\partial }{{\partial \theta_{i} }}L\left( {\theta \left| X \right.} \right) = 0,\quad i = 1,2, \ldots k$$

### Performance comparison

The Friedman test is used to check for differences in performance across multiple trials to accurately compare the modelling performance of different probability distributions in different wind parks^33^. In particular, column effects are checked after adjusting with possible row effects. The significant level of the Friedman test was set as 0.01 in the present study.

- *H*_*0*_: The column data do not have a significant difference.
- *H*_*a*_: The column data have a significant difference.

The statistic *F* is shown as in (6):

6 $$F = \frac{12n}{{k\left( {k + 1} \right)}}\left[ {\sum\limits_{i = 1}^{k} {r_{i}^{2} - \frac{{k(k + 1)^{2} }}{4}} } \right]$$

where *k* is the number of columns, *r*_*i*_ is the mean value of row *i*. It follows ${\chi }_{(k-1)}^{2}$ under *H*_*0*_.

## Experiment setup and evaluation

### Estimation of PDF

We used 0.5 m/s as the bin size to create histograms of hourly wind speed throughout the whole year for the wind speed data from the NWP models at the five wind parks and the observed wind speed of the Fakken wind park. The MLE method was then used to estimate the parameters required to define each of the theoretical PDFs, as described in “Parametric estimation”. A one-sample Kolmogorov–Smirnov test (K–S test) was conducted to confirm whether the original wind speed data came from calculated ideal distributions by comparing the CDF of the original data and fitted ideal distributions.

Since the histogram is discrete, the kernel distribution is typically taken an empirical nonparametric PDF modelling method based on the original data. It harnesses the kernel functions (typically Gaussian function) to connect adjacent bins of the histogram to create continuous PDFs of the data. Unlike histograms, the kernel distribution approximates infinitesimal length sampling, thereby reducing sampling errors between each bin. Therefore, it can be considered a more real historical distribution of raw data. Graphically, we named this ‘PDF smoothing’, and it was defined by a kernel function *K*(·) and a bandwidth *d* in (7):

7 $${\widehat{f}}_{d}(x)=\frac{1}{nd}\sum_{i=1}^{n} K\left(\frac{x-{x}_{i}}{d}\right)$$

We conducted two separate modelling analyses—overall and interval wind speed PDF fitting—to achieve a better understanding of the probabilistic characteristic of wind. Based on five wind park features and wind turbine power curve characteristics of our six cases, we choose the wind speed interval related to the rated power, with the rated speed of 10 m/s and the cut-off speed of 20 m/s, which are typical parameters for commercial medium-size wind turbines.

### Performance evaluation criteria

The K–S test is a nonparametric statistical test based on cumulative distribution function that tests whether a distribution is different from a type of ideal distribution^34^. A nonparametric test is used to test a hypothesis^35^.

- *H*_*0*_: {X_1_, X_2_, …, X_*n*_} has a given continuous distribution.
- ${H}_{a}:$ At least one does not come from the given distribution.

The K–S test is constructed from the statistic in Eq. (8):

8 $$D=\underset{x}{sup} \left|{F}_{0}(x)-F(x)\right|$$

where ${F}_{0}\left(x\right)$ represents CDF of the given ideal distribution, and $F(x)$ is CDF of {X_1_, X_2_, …, X_*n*_}. The test statistic is compared to critical values from the theoretical distribution of the Brownian bridge (If a Brownian motion, which is the random motion of particles suspended in a medium, starts at a certain point and returns to the starting point at the end, the process is called Brownian bridge)^36^.

To evaluate and compare the different examined performance of PDFs for modelling the wind speed, the mean absolute error (MAE) and root mean square error (RMSE) were used to calculate the probability density difference between parametric ideal distributions and the original PDF smoothing with speed unit of 0.01 m/s. Both are negatively oriented metrics, indicating that the smaller values are related to better performance. The MAE and RMSE determine the accuracy of a model by calculating averages of the absolute and square difference between the histogram-based PDF from the NWP and observed data and different theoretical PDF models, as expressed in Eqs. (9) and (10). The RMSE assigns a higher weight to larger errors due to the square calculation, which penalises more significant model errors and indicates whether the model has a significant error variance^37^. Hence, the MAE and RMSE provide a comprehensive representation of a model's performance

9 $$MAE=\frac{{\sum }_{i=1}^{n}\left|{modeling}_{i}-{smoothing}_{i}\right|}{n}$$

10 $$RMSE=\sqrt{\frac{{\sum }_{i=1}^{n}{\left({modeling}_{i}-{smoothing}_{i}\right)}^{2}}{n}}$$

## Results and discussion

### PDF modelling graph

**Figure 1:**
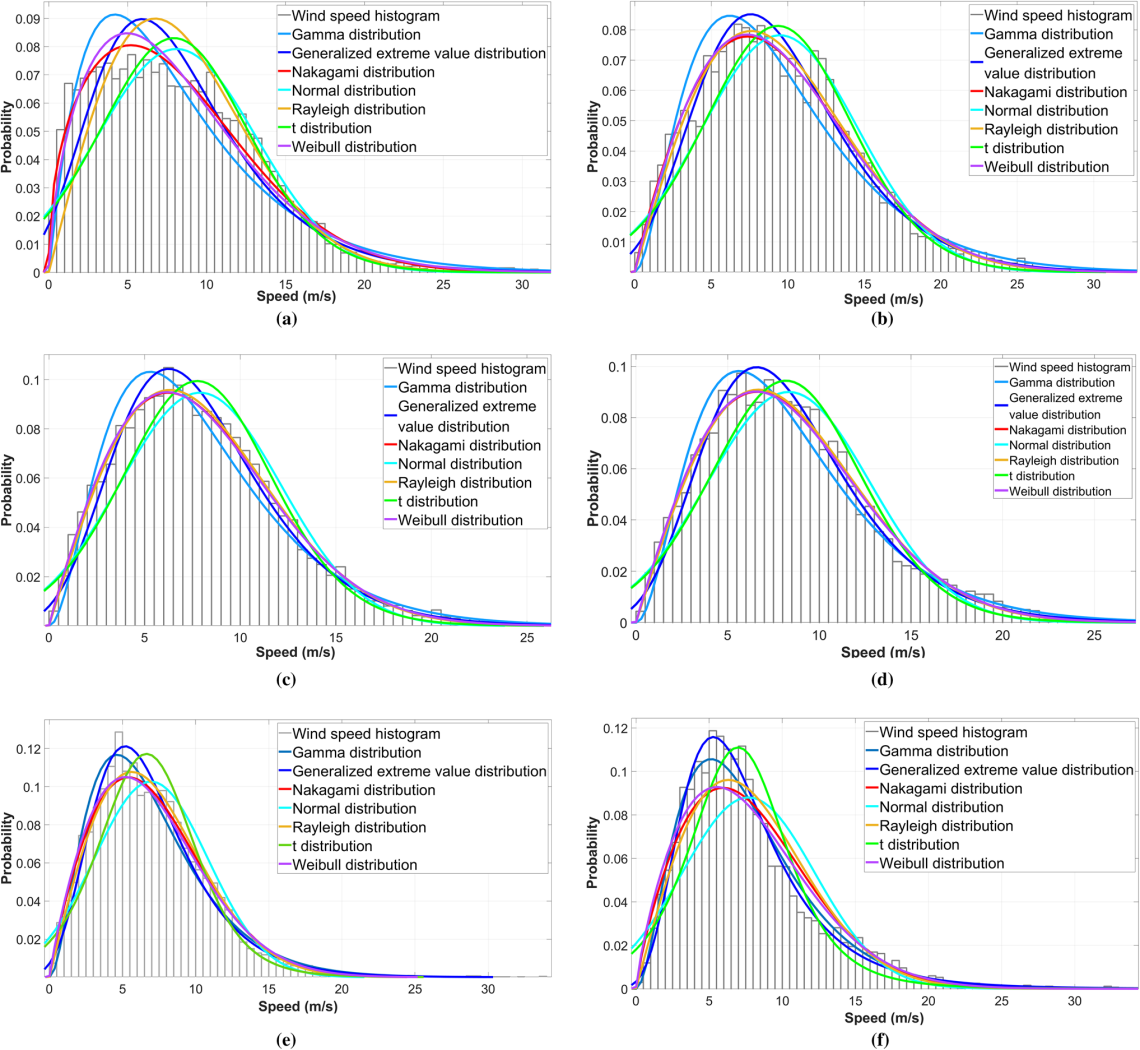
The estimated PDFs curve graphs for NWP model data of five sites and measurements from Fakken wind park (NWP: (**a**) Nygårdsfjellet, (**b**) Raggovidda, (**c**) Kjøllefjord, (**d**) Havøygavlen, (**e**) Fakken, Measurements: (**f**) Fakken).

Histograms and PDFs graphs are shown in Fig. 1 to show the estimated ideal PDFs with the MLE method for different cases. Discontinuous histograms are represented by bar charts (for clarity, we ignored the kernel distribution curves in these figures), and the fitted probability distribution curves are shown in different colours. As can be seen, although the wind speed distributions of different wind parks varied, they had some similarities. It is also clear that different probabilistic models provide differing fits to wind speeds. In particular, when comparing (e) and (f), the actual wind speed is more centrally concentrated and possesses a thicker tail. Due to the scarcity of data, this phenomenon could only be considered empirical for the Fakken site.

### K–S test

The K–S test is a rigorous statistical test. Passing this test indicates that there is no statistically significant difference between the PDFs of original data and ideal distributions. The null hypothesis in the present study was that wind speed data fit a mentioned ideal distribution; however, they also could not originate from such an ideal distribution. The significance level was set at 1%, and the results of the K–S test are given in Table 4. 'Pass' means that the K–S test did not reject the null hypothesis, and 'Fail' indicates that the K–S test rejected the null hypothesis.

**Table 4:** The result of the K–S test.

Wind park	Gamma	GEV	Nakagami	Normal	Rayleigh	t	Weibull
Nygårdsfjellet	Fail	Fail	Fail	Fail	Fail	Fail	Fail
Raggovidda	Fail	Fail	Pass	Fail	Fail	Fail	Pass
Kjøllefjord	Fail	Fail	Pass	Fail	Pass	Fail	Pass
Havøygavlen	Fail	Pass	Pass	Fail	Pass	Fail	Pass
Fakken (NWP)	Fail	Fail	Fail	Fail	Fail	Fail	Fail
Fakken (MEASURE)	Fail	Pass	Fail	Fail	Fail	Fail	Fail

As is shown, none of the distributions could pass all the K–S tests at the 1% significance level. In addition, the Gamma, normal and t distributions failed the tests in all cases. Meanwhile, the Nakagami and Weibull distributions passed the test for three of the NWP wind data sets, while no distributions passed the tests for Nygårdsfjellet. Regarding the comparison of the PDF modelling between the NWP and observed wind speed of Fakken, all distributions failed the tests for Fakken (NWP), and only the GEV distribution passed the test for Fakken (MEASURE). Therefore, the different probabilistic models each have particular strengths that vary according to wind park and data types.

### Overall wind speed PDF modelling

Table 5 shows the calculated parameters by MLE of different PDF models.

**Table 5:** The parameters of fitted PDFs.

	Gamma	GEV	Nakagami	Normal	Rayleigh	t	Weibull
Nygardsfjellet	2.07; 3.91	5.80; 4.10; − 0.02	0.72; 90.93	8.10; 5.04	6.74	17.87	1.62; 9.02
Raggovidda	2.90; 3.27	7.26; 4.10; − 0.07	0.94; 116.08	9.50; 5.10	7.62	20.66	1.93; 10.69
Kjøllefjord	3.04; 2.60	6.04; 3.10; − 0.05	0.97; 80.15	8.10; 5.04	6.33	16.67	1.96; 8.91
Havoygavlen	3.07; 2.71	6.37; 3.10; − 0.05	0.98; 89.14	8.34; 4.43	6.68	16.84	1.96; 9.40
Fakken (NWP)	2.99; 2.32	5.18; 3.10; 0.00	0.94; 63.37	6.95; 3.89	5.63	7.48	1.87; 7.83
Fakken (MEASURE)	3.01; 2.56	5.54; 3.10; 0.08	0.90; 79.62	7.69; 4.53	6.31	4.26	1.80; 8.68

The parameters are shown with the form in Table 2 in order corresponding to each PDF.

**Figure 2:**
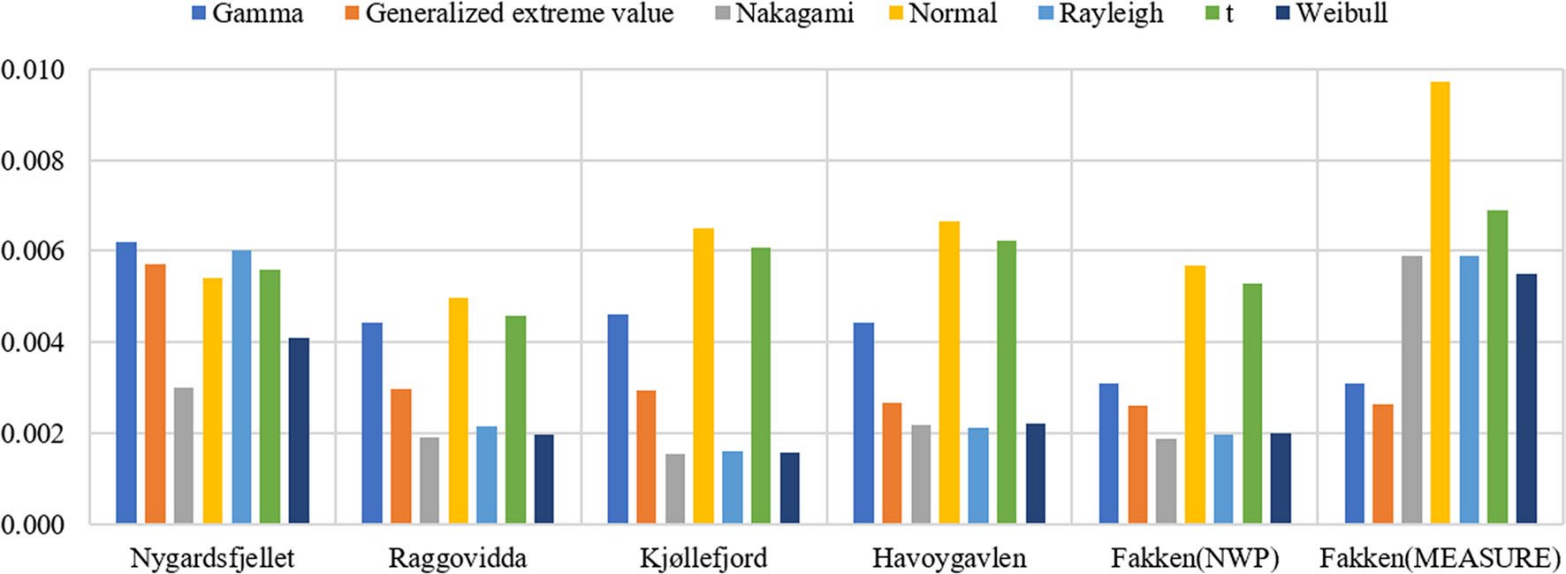
The overall MAE of wind speed PDFs for NWP data from five sites and measurements from Fakken.

The overall MAE of wind speed PDF fitting for the NWP model from five sites and measurements from Fakken is given in Fig. 2. For the NWP wind speed data, the Nakagami distribution generally had a lower MAE than the other distributions. One exception to this is Havøygavlen, in which the Rayleigh distribution performed the best. The normal t distributions had the worst performance in terms of MAE. For the Nakagami distributions for NWP wind speed from different wind parks, the MAEs of Kjøllefjord and Fakken (which are characterised by rougher terrain) were lower compared with the other wind parks. For the observed wind speed data fitting of Fakken, the GEV distribution had the lowest MAE; here, the edge was even more significant than the Nakagami distribution for Fakken NWP data modelling. In addition, the overall MAE of Fakken measured wind speed modelling was much larger than for the NWP data of Fakken.

**Figure 3:**
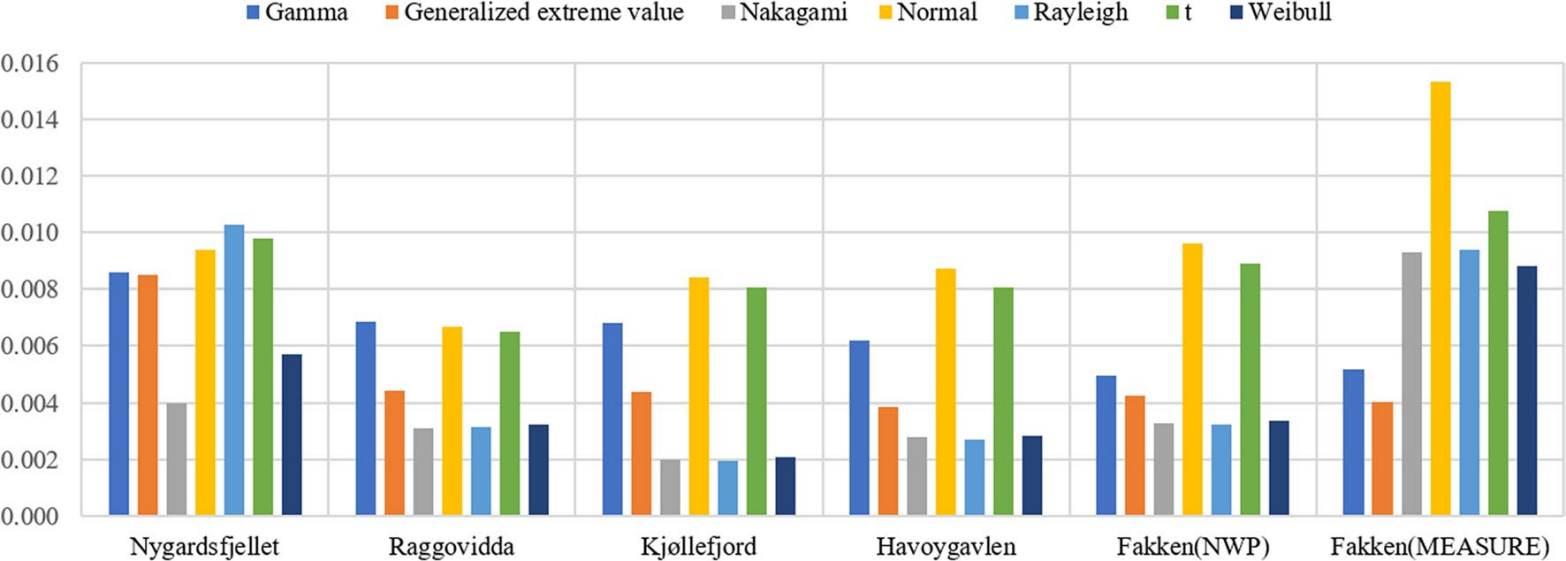
The overall RMSE of wind speed PDFs for NWP data from five sites and measurements from Fakken.

The overall RMSE of the overall wind speed PDF for the NWP model of five sites and measurements of Fakken wind park is displayed in Fig. 3. In relation to NWP wind speed data, the Nakagami and Rayleigh distributions showed a low RMSE between the histogram and parameterised PDFs, except for Nygårdsfjellet. The overall RMSE of the normal and t distributions was relatively high. Kjøllefjord had the lowest RMSE in the Nakagami and Rayleigh distribution. In terms of the RMSE of wind speed measured data from Fakken, the results were similar to the overall MAE results.

Friedman tests for the overall MAE and RMSE of wind speed PDF modelling for the NWP data from five sites were conducted to determine whether there were statistical differences between different probability distribution modelling approaches (effect of distributions) and whether there were statistical differences in the probabilistic modelling results for different wind parks (effect of parks). All the *p* values surpassed the confidence level of 0.01; therefore, the Friedman test’s null hypothesis was not rejected. The results are shown in Table 6.

**Table 6:** The *p* values of the Friedman test for overall wind speed modelling.

	Effect of distributions	Effect of parks
MAE	0.0011	0.0525
RMSE	0.0014	0.0029

### Interval wind speed PDF modelling

**Figure 4:**
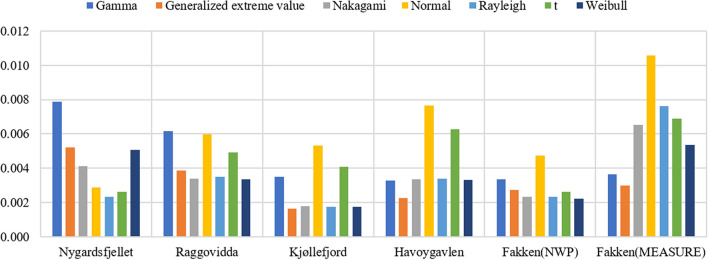
The MAE of interval wind speed PDFs for NWP data from five sites and measurements from Fakken.

The MAE of interval wind speed PDF is shown in Fig. 4. The results showed some differences from their counterparts in the overall modelling. For the NWP wind speed data, the optimal for Nygårdsfjellet was obtained with the Rayleigh distribution. The Weibull distribution had a slight advantage over the Nakagami and Rayleigh distributions, while the normal distribution showed the worst MAE performance on the whole. The MAE of the Weibull distributions for Kjøllefjord were the smallest out of the five wind parks. Regarding the distributions of measured wind speed of Fakken, the overall MAE was much larger than for the Fakken NWP data; further, the GEV produced the lowest MAE.

**Figure 5:**
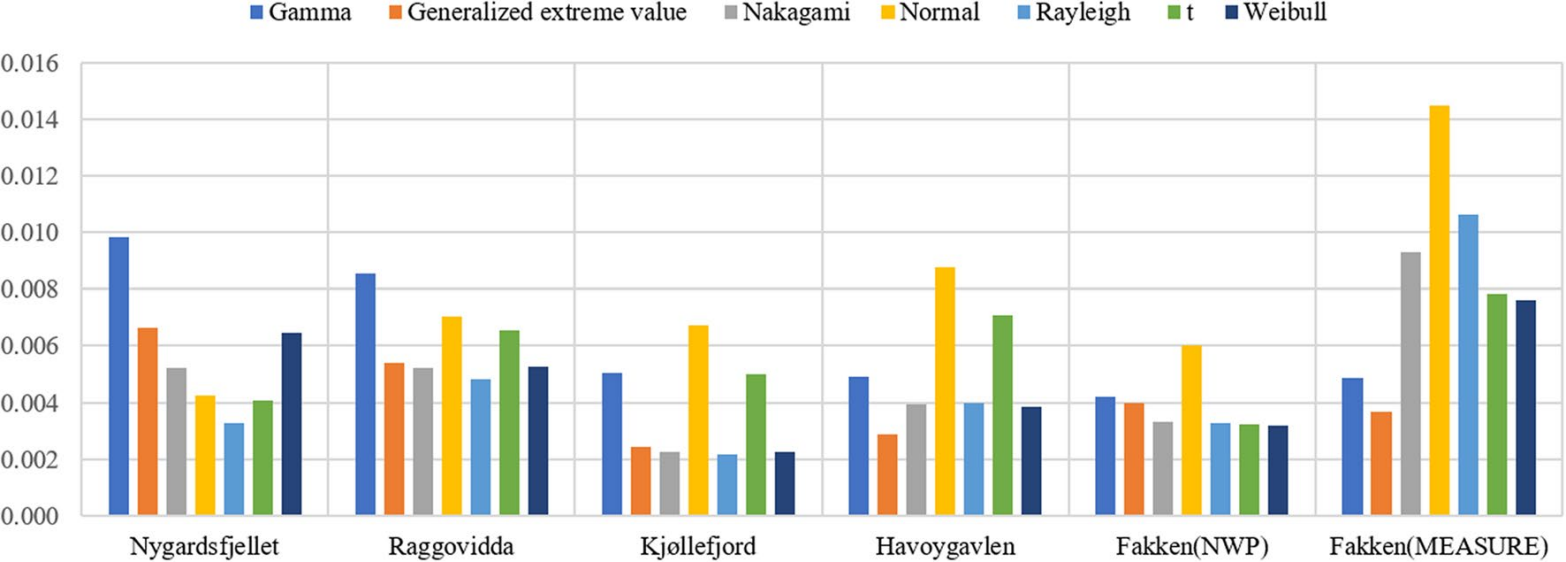
The RMSE of interval wind speed PDFs for NWP data from five sites and measurements from Fakken.

The RMSE of the interval wind speed PDF is shown in Fig. 5. The results were similar to the MAE evaluation of interval modelling. For the NWP wind speed data, the Rayleigh distribution was superior to other distributions for Nygårdsfjellet. The Nakagami, Rayleigh and Weibull distributions had almost the same RMSEs for the remaining four wind parks, while for the RMSE of the observed data from Fakken, the GEV distribution still won.

Similarly, differences in interval wind speed from NWP probabilistic modelling between wind parks were tested, and the results are given in Table 7. All *p* values exceeded the confidence level of 0.01, which suggests that there are statistical differences between different probability distribution modelling methods and in the probabilistic modelling of different wind parks.

**Table 7:** The *p* values of the Friedman test for interval wind speed modelling.

	Effect of distributions	Effect of parks
MAE	0.0717	0.05
RMSE	0.0156	0.0134

### Discussion

In summary, the Nakagami distribution is recommended as the preferred model for the PDF of NWP wind speed data, as it showed excellent and consistent performance. The Nakagami and Weibull distributions could generally capture essential characteristics of the historical distributions of wind speed for both NWP model data by K–S tests. The GEV distribution could describe the statistics of the observed wind data in the examples we used. Moreover, PDF modelling for the NWP wind speed was more accurate compared with actual measurements of wind speed.

In terms of evaluating the NWP wind speed, for the overall wind speed PDF modelling performance, the Nakagami and Weibull distributions showed a good fit for all five wind parks’ overall PDFs of NWP wind speed data. In comparison, the Rayleigh distribution provided a favourable overall fit for all except Nygårdsfjellet. The Nakagami and Rayleigh distributions also performed excellently for the wind speed interval modelling. Generally, we made a more precise PDF fitting for NWP wind speed data from Kjøllefjord than for other wind parks both in overall and interval wind speed modelling. This was unexpected, as Kjøllefjord has the highest RIX (10–20) of all of them. In addition, Havøygavlen and Fakken, with RIXs (5–10), were also fitted better than Nygårdsfjellet and Raggovidda with RIXs (0–5). Further research is needed because it is generally thought that the more complex the terrain is, the more difficult it is to use NWP to forecast the wind speed^38^.

For the actual observed wind data from Fakken, the GEV distribution was superior to all other distributions both in overall and interval wind speed modelling and should be used to assess wind speed in this area. The differences between NWP wind data and real measurements of Fakken can be summarised as follows. First, referring to Table 2, the observed speed had a higher mean value, standard deviation, coefficient of variation and skewness, though lower kurtosis meant that the measurements varied more from the normal distribution and had a lighter distribution tail than the NWP data. Second, the best distributions were the Nakagami and Generalised extreme value distribution, respectively. The Weibull distribution, which is typically used for wind speed modelling, was inferior to these two methods in our cases.

## Conclusions

The statistical characteristics of wind speed are essential for the practical assessment of wind energy potential and the sustainable design of wind parks. In the present study, we concentrated on probabilistic modelling of NWP wind speed for five wind parks in the Norwegian Arctic region and one observed wind speed for one of them. Our results are based on 1 year of data, and a longer period is needed to conduct a wind resource assessment of a potential wind park site. Using longer time series would provide a better estimate of the wind speed distribution for NWP and measurements and a better understanding of rare extreme high wind events. The results of the present study indicated that, for wind resource assessments in complex terrain, the Nakagami and Generalised extreme value distributions are recommended as the preferred models for the PDF of NWP and observed wind speed, respectively, as they showed excellent and consistent performance. In addition, the probabilistic models that reasonably describe interval wind speed differ from those of overall wind speed due to the nature of the wind: the former corresponds more to the right-side properties of the probability distribution functions.

Based on the results of this study, the following policy recommendations are provided.

1. Different probabilistic modelling approaches should be considered when conducting wind resource potential assessments to achieve more accurate estimations.
2. The wind speeds of neighbouring regional wind parks are characterised by similarities and synergies partly due to the probabilistic models that accurately describe them are identical. But in wind engineering reality, Topography, meteorology, turbine selection and layout etc. all affect the power generation of a wind park. Therefore, the possibility of simultaneous intermittency of these wind parks must be considered when exploiting wind power in the area. Reasonable compensations for other energy sources are required.
3. Compared with observed wind speeds, numerical predicted speeds can be better described by probabilistic models; therefore, when using numerical meteorology to assess wind resources, more consideration should be given to extreme wind events. Some allowance may be made for errors in wind energy project development.

## Data Availability

The NWP data is public available from The Norwegian Meteorological Institute. The measured wind data from Fakken wind park is the property of the power company Troms Kraft AS.
